# Compound A attenuates toll-like receptor 4-mediated paclitaxel resistance in breast cancer and melanoma through suppression of IL-8

**DOI:** 10.1186/s12885-018-4155-6

**Published:** 2018-02-27

**Authors:** Rochanawan Sootichote, Peti Thuwajit, Ekapot Singsuksawat, Malee Warnnissorn, Pa-thai Yenchitsomanus, Suthinee Ithimakin, Jomjit Chantharasamee, Chanitra Thuwajit

**Affiliations:** 1 Graduate Program in Immunology, Department of Immunology, Faculty of Medicine Siriraj Hospital, Mahidol University, Bangkok, 10700 Thailand; 2 Department of Immunology, Faculty of Medicine Siriraj Hospital, Mahidol University, Bangkok, 10700 Thailand; 3 Department of Pathology, Faculty of Medicine Siriraj Hospital, Mahidol University, Bangkok, 10700 Thailand; 4 Siriraj Centre of Research Excellence for Cancer Immunotherapy, Division of Molecular Medicine, Department of Research and Development, Faculty of Medicine Siriraj Hospital, Mahidol University, Bangkok, Thailand; 5 Division of Oncology, Department of Internal Medicine, Faculty of Medicine Siriraj Hospital, Mahidol University, Bangkok, 10700 Thailand

**Keywords:** TLR4, Paclitaxel, Breast cancer, Melanoma, Tumor microenvironment, Compound A

## Abstract

**Background:**

Paclitaxel (PTX) is a potent anti-cancer drug commonly used for the treatment of advanced breast cancer (BCA) and melanoma. Toll-like receptor 4 (TLR4) promotes the production of pro-inflammatory cytokines associated with cancer chemoresistance. This study aims to explore the effect of TLR4 in PTX resistance in triple-negative BCA and advanced melanoma and the effect of compound A (CpdA) to attenuate this resistance.

**Methods:**

BCA and melanoma cell lines were checked for the response to PTX by cytotoxic assay. The response to PTX of TLR4-transient knockdown cells by siRNA transfection was evaluated compared to the control cells. Levels of pro-inflammatory cytokines, IL-6 and IL-8, and anti-apoptotic protein, XIAP were measured by real-time PCR whereas the secreted IL-8 was quantitated by ELISA in TLR4-transient knockdown cancer cells with or without CpdA treatment. The apoptotic cells after adding PTX alone or in combination with CpdA were detected by caspase-3/7 assay.

**Results:**

PTX could markedly induce *TLR4* expression in both MDA-MB-231 BCA and MDA-MB-435 melanoma cell lines having a basal level of TLR4 whereas no significant induction in *TLR4*-transient knockdown cells occurred. The si*TLR4*-treated BCA cells revealed more dead cells after PTX treatment than that of mock control cells. *IL-6*, *IL-8* and *XIAP* showed increased expressions in PTX-treated cells and this over-production effect was inhibited in TLR4-transient knockdown cells. Apoptotic cells were detected higher when PTX and CpdA were combined than PTX treatment alone. Isobologram exhibited the synergistic effect of CpdA and PTX. CpdA could significantly decrease expressions of *IL-6*, *XIAP* and *IL-8*, as well as excreted IL-8 levels together with reduced cancer viability after PTX treatment.

**Conclusions:**

The acquired TLR4-mediated PTX resistance in BCA and melanoma is explained partly by the paracrine effect of IL-6 and IL-8 released into the tumor microenvironment and over-production of anti-apoptotic protein, XIAP, in BCA cells and importantly CpdA could reduce this effect and sensitize PTX-induced apoptosis in a synergistic manner. In conclusion, the possible impact of TLR4-dependent signaling pathway in PTX resistance in BCA and melanoma is proposed and using PTX in combination with CpdA may attenuate TLR4-mediated PTX resistance in the treatment of the patients.

**Electronic supplementary material:**

The online version of this article (10.1186/s12885-018-4155-6) contains supplementary material, which is available to authorized users.

## Background

Breast cancer (BCA) is a common cancer worldwide and the first ranked cancer in Thai women [1]. Taxanes-based chemotherapy provides good outcomes of both early and advanced BCA and has been preferentially recommended to treat both advanced and metastatic BCA patients [2]. Standard chemotherapy is the only systemic treatment in triple-negative BCA. Paclitaxel (PTX), one of the taxanes, is widely used in advanced cancer; however, anthracycline-pretreated BCA patients were up to 74% PTX resistant [3]. Melanoma is the most dangerous type of skin cancer with annually increasing incidence and death rates. PTX is used as a second line drug of metastatic melanoma with the evidence of resistance; and a group of studies have proposed the way to increase sensitivity of PTX in melanoma [4, 5].

Toll-liked receptors (TLRs), classified as the damage-associated molecular pattern (DAMP) receptors, have been implicated in the development of several cancers including BCA. TLR4 expression levels increased in BCA tissues compared to normal breast tissues and activation of TLR4 by lipopolysaccharide (LPS) mediated the cancer cell migration in vitro [6]. Ligand-TLR ligation triggers downstream signaling pathways for production of several pro-inflammatory mediators during inflammation, some of which play a major role in carcinogenesis and tumor progression [7]. Interestingly, TLR4 has been reported as the receptor of PTX [8]. Moreover, TLR4 has been involved in its impact on PTX resistance causing the increase of pro-inflammatory cytokines and their receptors to favor cancer aggressiveness and chemoresistance in ovarian cancers [9–12]. Activation of the TLR4 pathway by LPS in BCA cells promoted cell migration and probably involved cancer metastasis [13]. The evidences in melanoma cells has been reported that TLR4-MyD88-ERK signaling may be a novel target for reversing chemoresistance to PTX [5].

Compound A (CpdA) or 2-(4-acetoxyphenyl)-2-chloro-N-methylethylammonium chloride, a non-steroidal dissociated glucocorticoid receptor (GR) ligand, can mediate gene-inhibitory effects via GR activation resulting in disruption of NF-κB and AP-1 which importantly generates the expression of pro-inflammatory cytokine mediators [14]. CpdA could modulate cancer aggressiveness which abrogated NF-κB and AP-1 activation resulting in induced pro-apoptotic protein expressions such as BCL2L11 or Bim and Bik together with p53 and inhibited pro-inflammatory cytokine expression [14–16]. TLR4 ligation by PTX could activate downstream signaling via NF-κB and MAPK that promote pro-inflammatory mediators i.e. IL-6, IL-8, MCP-1, VEGF and XIAP, the functions of which have been reported to enhance cancer progression and drug resistance [9–11]. A combination of CpdA and PTX supporting the reduction of inflammation and cancer aggressiveness, however, has not been demonstrated in BCA and melanoma.

This study aims to explore role of TLR4 in PTX resistance of triple-negative BCA and melanoma. Cells with and without TLR4 were studied and correlated with their responses to PTX. Furthermore, TLR4-activated pro-inflammatory mediators, in particular IL-6 and IL-8, and anti-apoptotic protein XIAP were investigated for their potential impact in PTX resistance. The impact of CpdA to PTX resistance of the two cancer cells through TLR4 mediated production of these cytokines was demonstrated. The synergistic effect of CpdA and PTX to induce cancer cell apoptosis was revealed. This knowledge might then be proposed for the potential of using TLR4 in the prediction of PTX response; and targeting the PTX-TLR4 mediated signaling pathway in cancer cells by CpdA could then be a challenge to alleviate PTX resistance in triple-negative BCA and melanoma.

## Methods

### Human BCA and melanoma cell lines

Human BCA cell lines: MCF-7 (ATCC, #HTB-22) and MDA-MB-231 (ATCC, #HTB-26); and human melanoma cell line: MDA-MB-435 (ATCC, #HTB-129) were obtained from Chulabhorn Research Institute, Bangkok. They were grown in Dulbecco’s modified Eagle’s medium (DMEM) (Gibco, Invitrogen, Carlsbad, CA) supplemented with 10% (*v*/v) fetal bovine serum (FBS) (Gibco). Cells were maintained in an incubator at 37 °C with 5% CO_2_.

### Quantitative real-time PCR

Total RNA was extracted by PerfectPure® RNA Cultured Cell Kit (5 PRIME, Gaithersburg, MD) and reverse transcribed using the Superscript® III First-Strand synthesis system (Invitrogen, Carlsbad, CA) according to manufacturer’s instructions. Primers were designed based on NCBI database. The primer sequences and the individual PCR product sizes are demonstrated in Table 1. Targets were amplified for 40 cycles with denaturation at 95 °C for 15 s and annealing at 60 °C for 15 s and extension at 72 °C for 45 s. For *MyD88*, cDNA was amplified for 60 cycles of denaturation at 95 °C for 15 s and annealed at 60 °C for 15 s using Lightcycler® 480 software version 1.5 (Roche Applied Science, Mannheim, Germany). Gene expressions were normalized with β-actin (*ACTB*) and determined by the ∆C_T_ method.

**Table 1:** Sequences of primers for real-time PCR

Gene	Accession no.	Primer (5′-- > 3′)		Size (bp)
*ACTB*	NM_001101.3	F	CACACTGTGCCCATCTACGA	162
		R	CTCCTTAATGTCACG CACGA	
*TLR4*	NM_138554	F	TCACAGAAGCAGTGAGGATGAT	140
		R	AAGTAATATTAGGAACCACCTCCA	
*MyD88*	NM_002468	F	TGCAGAGCAAGGAATGTGAC	153
		R	GGTTGGTGTAGTCGCAGACA	
*IL-6*	NM_000600	F	CGGGAACGAAAGAGAAGCTCTA	68
		R	GGCGCTTGTGGAGAAGGAG	
*IL-8*	NM_000584	F	GCCAACACAGAAATTATTGTAAAGCTT	112
		R	AATTCTCAGCCCTCTTCAAAAACTT	
*XIAP*	NM_001167	F	CCATGGCAGATTATGAAGCA	176
		R	TTGTTCCCAAGGGTCTTCAC	

### Transfection of siTLR4 to transient knock down of TLR4 in cancer cells

The 1 × 10^5^cells of MDA-MB-231 and MDA-MB-435 cells were cultured in 6-well plates and grown in DMEM without antibiotics at 37 °C with 5% CO_2_ for 24 h. Then the cells were transfected with 2.5 μg and 1.25 μg of siRNA targeted to human TLR4 (Cat.no. 40260, Santa Cruz Biotechnology Inc., Dallas, TX) for MDA-MB-231 and MDA-MB-435 cells using Lipofectamine™ 2000 (Invitrogen) in the serum-reduced Optimem® (Invitrogen).

### Immunocytochemistry of TLR4 on cancer cells

Cancer cell lines were plated in 96-well plate and fixed with 4% (*w*/*v*) paraformaldehyde in 0.1 M Na_2_HPO_4_ and 0.1 M NaH_2_PO_4_.H_2_0. Cells were blocked with 1% (w/v) bovine serum albumin (BSA) and incubated with 1:5 goat anti-human TLR4 polyclonal antibody (AF1478, R&D Systems, MN) for 2 h at 37^๐^C and subsequently stained with 1:500 donkey anti-goat IgG-Alexa® 488 (cat. no. A11055, Invitrogen, CA) for 1 h with light protection. Nucleus was counterstained simultaneously with 1:1000 Hoechst® 33,258 (Invitrogen, CA). TLR4 localization was observed by the inverted microscope model IX71 (Olympus America, NY).

### Western blot analyses of TLR4 in BCA cells with or without transfection with siTLR4

Human BCA cell lines were trypsinized with 0.05% (*v*/v) trypsin-EDTA (Invitrogen) and washed twice with 1X phosphate buffer saline (PBS). Cells were lysed completely in 100 μl of sample buffer (0.25 M Tris-HCl pH 6.8, 20% glycerol, 4% sodium dodecyl sulfate (SDS), 0.05% (*w*/*v*) bromophenol blue, β-mercaptoethanol and distilled water) and heated in a boiling water bath for 10 min and cell debris cleared by centrifugation at 8,000X g for 1 min. Protein samples were separated in 8% SDS-polyacrylamide gel electrophoresis (SDS-PAGE) and transferred to polyvinylidene fluoride (PVDF) membranes by TE 70 Semi-Dry transfer units (GE Healthcare, Buckinghamshire, UK) for 1.5 h. The membranes were blocked in a blocking solution [5% (w/v) skim milk in 0.1% (*v*/v) Tween 20 in TBS-T] overnight at 4^๐^C. For detecting TLR4 protein, the blots were probed by incubation for 1 h at room temperature with rabbit anti-human TLR4 polyclonal antibody (1: 1000) (sc-10,741, Santa Cruz Biotechnology Inc.) in a blocking reagent for 1 h. The blots were then washed in TBS-T before being incubated with 1:1500 dilution of goat anti-rabbit IgG HRP (ab6721, Abcam, Cambridge, MA) for 1 h. For β-ACTIN detection, mouse anti-human β-actin monoclonal antibody (1: 10,000) (sc-47,778, Santa Cruz Biotechnology Inc.) and either HRP-conjugated rabbit anti-mouse IgG antibody (61–6020, Invitrogen) or HRP-conjugated goat anti-mouse IgG antibody (62–6620, Invitrogen) diluted 1:2000 in blocking solution were used for primary and secondary antibodies. The protein bands were visualized by SuperSignal® West Pico chemiluminescent substrate (Pierce, Thermo Scientific, Rockford, IL) and exposed to film or G:BOX Chemi XR5 GelDoc (Syngene, Suite L Frederick, MD). The band intensities were measured by ImageJ® software [17]. The level of β-ACTIN was used as an internal control to ensure equal amounts of loading proteins.

### Cytotoxic assay

BCA cell lines were cultured at the density 1.3ˣ10^4^ cells per well in 96-well plates. After 24 h, various doses of PTX (T7191, Sigma Aldrich, St. Louis, MO) in complete medium were added into each well for individual drug concentrations with 100 μl of the final volume. Cells were further incubated with the drug for 24 h. At the end of the experiment, cell viability was measured using trypan blue staining. The number of cells in each drug treatment condition by compared to those without drug in the medium as the negative control to determine the LC_50_. The assay was performed as independent duplicates.

Cells were seeded at the density 1.3ˣ10^4^ cells per well in 96-well plates. After a 24 h culture period to permit cells completely adhere to the culture plate, different concentrations of PTX or CpdA [Laboratory of Eukaryotic Gene Expression & Signal Transduction (LEGEST), Department of Physiology, Ghent University, Belgium] kindly donated by Professor Guy Haegeman were dissolved in complete medium and were added into wells for individual drug concentrations with 100 μl as the final volume. Cells were further incubated with the drug for 24 h. At the end of the experiment, cell viability was measured by using trypan blue staining and MTS assay (Promega, Mandison, WI) following manufacturer’s instruction. The absorbance was detected at 490 nm with a microplate reader. Cells cultured in the complete media were used as a control. The assays were performed in triplicate.

### Evaluation for synergistic combination

1.3ˣ10^4^ cells of either MDA-MB-231 or MDA-MB-435 were seeded into 96-well plate. After 1-day, different concentrations of PTX or CpdA in complete medium was added into each well for individual drug concentration with 100 μl of the final volume. Cells were further incubated for 24 h. At the end of the experiment, cell viability was measured and the number of cells in each drug treatment condition was compared to those without drug in the medium as negative control. LC_50_ against PTX and CpdA were analyzed for synergistic effect by plotting isobologram. The assay was performed in triplicate.

### Apoptosis assay

Cancer cell lines including MDA-MB231 BCA and MDA-MB435 melanoma were cultured in 96-well plates for 24 h. Caspase-3/7 Green Reagent for Apoptosis assay (Essen BioScience, Ann Arbor, MI) was added following the manufacturer’s instruction together with PTX or CpdA or the combined treatment. The live-cell analysis was performed in the IncuCyte ZOOM™ (Essen BioScience, Ann Arbor, MI) to detect apoptotic cells by scanning every 3 h for a total of 48 h and the data was analyzed by the IncuCyte ZOOM software.

### Enzyme-linked immunosorbent assay (ELISA)

Conditioned medium was collected from MDA-MB-231 and MDA-MB-435 BCA cell lines treated with either PTX or CpdA. Concentrations of IL-8 were investigated by Human IL-8/NAP-1® Instant ELISA (eBioscience, Vienna, Austria) following the instruction manual. The results were measured on a Biochrom Anthos® 2010 microplate reader (Biochrom Ltd., Cambridge, UK). All experiments were performed in duplicate and reproduced twice.

### Statistical analysis

The differences of gene and protein expressions between test and control samples were determined by Student’s t-test. All quantified data were presented as mean ± standard deviation (SD). LC_50_ against PTX was performed by GraphPad® Prism 5. The *p*-values of equal or less than 0.05 were considered to be statistically significant.

## Results

### TLR4 mediates resistance to PTX-induced BCA and melanoma cell death

**Fig. 1:**
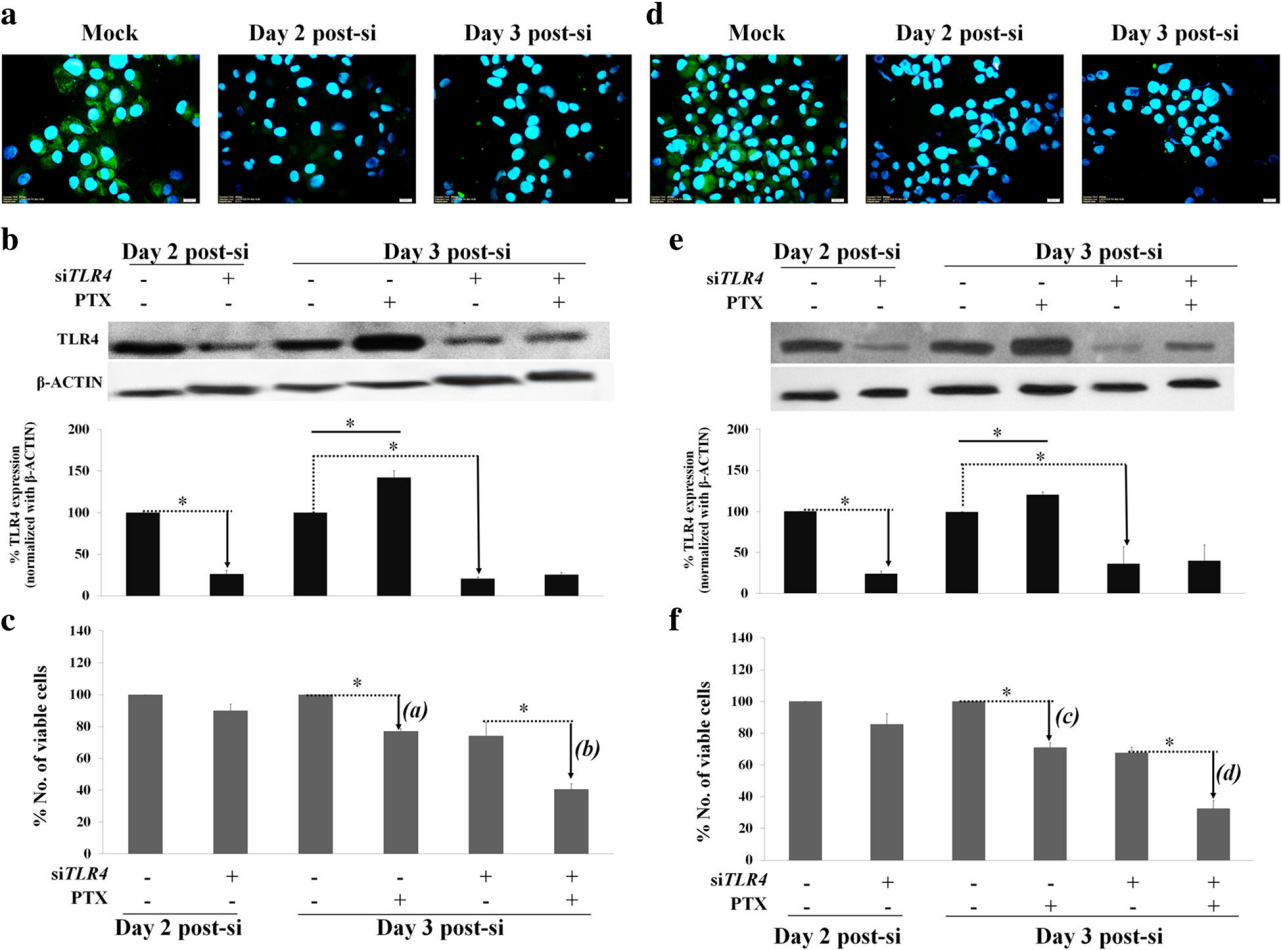
Transient knockdown of *TLR4* in BCA cell lines and the response to PTX. Effect of si*TLR4* in (**a**) MDA-MB-231 and (**d**) MDA-MB-435 cells. The level of TLR4 detected by western blot analyses in parental and si*TLR4*-treated (**b**) MDA-MB-231 and (**e**) MDA-MB-435 cells treated with or without PTX. β-ACTIN was used as an internal control. The bands were quantified by ImageJ® software. The percentage of viability after exposure to PTX in parental and si*TLR4*-treated MDA-MB-231 (**c**) and MDA-MB-435 (**f**) cells. The percentage of viable mock cells without PTX treatment was assumed to be 100%. Bars represent mean ± SD of independent duplicate experiments. *represents *p*-value < 0.05

MDA-MB-231, MDA-MB-435 and MCF-7 cell lines had different intrinsic TLR-4 levels (Additional file 1: Figure S1A and B). Total TLR4 levels produced from MDA-MB-231 and MDA-MB-435 were higher than that in MCF-7 (Additional file 1: Figure S1A). Membranous TLR4 confirmed the presence of this membrane receptor in all cell lines (Additional file 1: Figure S1B). MyD88 was expressed in all three cell lines with the highest level in MDA-MB-435 melanoma cells (Additional file 1: Figure S1C). Notably, different responses to PTX were detected in MDA-MB-231 and MDA-MB-435 cells with LC_50_ values of 8.8 μM and 0.42 μM, (Additional file 1: Figure S1D and E). The siRNA against *TLR4* was transfected into MDA-MB-231 and MDA-MB-435 cells using different concentrations: 2.5 μM and 1.25 μM. At day 2 post-si*TLR4* transfection (post-si), the reduction of membranous TLR4 detected by immunocytochemistry staining was detected in MDA-MB-231 (Fig. 1A) and MDA-MB-435 cells (Fig. 1D) and this transient knock down effect was sustained to day 3 post-si in both cells. The western blot results confirmed the significant reduction of TLR4 levels of around 60–80% at days 2 and 3 post-si (Fig. 1B and E). Interestingly, PTX treatment could significantly upregulate *TLR4* expression in both cancer cell lines whereas there were no significant alterations of TLR4 levels in TLR4-deficient cancer cell lines after PTX treatment (Fig. 1B and E).

The similar percentages of viable cells was observed in cells with or without si*TLR4* transfection represented no cytotoxic effect of the si*TLR4* to MDA-MB-231 and MDA-MB-435 (Fig. 1C and F). Notably, si*TLR4* transfection had too much toxic to MCF-7 cells (data not shown). Hence, MDA-MB-231 and MDA-MB-435 were used in the further experiment. After PTX treatment for 24 h, around 20% of parental cancer cells [Fig. 1C, *(a)*] were dead whereas si*TLR4*-treated cancer cells showed nearly 30% cell death [Fig. 1C, *(b)*]. Similarly, these results were exhibited in MDA-MB-435 melanoma cells that PTX-mediated resistance to PTX-induced cell death in parental cancer cells was more than that *TLR4*-deficient BCA cells [Fig. 1F, *(c)* and *(d)*]. In other words, cells having high levels of TLR4 had higher percentages of viable cells after exposure to the PTX anticancer drug.

### PTX induces TLR4-mediated IL-6, IL-8 and XIAP expressions and CpdA attenuates this effect

**Fig. 2:**
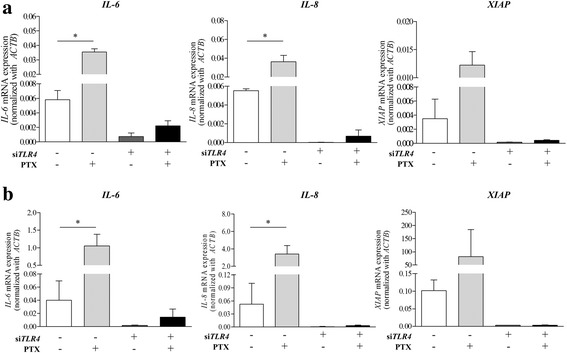
Effect of PTX on IL-6, IL-8 and XIAP expressions in BCA cells. **a** MDA-MB-231 and (**b**) MDA-MB-435 treated with or without si*TLR4*. Bars represent mean ± SD of independent duplicate experiments. *represents *p*-value < 0.05

PTX significantly augmented *IL-6* and *IL-8* expressions in MDA-MB-231 BCA (Fig. 2a) and in MDA-MB-435 melanoma cells (Fig. 2b) whereas *XIAP* mRNA was also induced by PTX but was not statistically significant. TLR4-transient knockdown cells had a reduction of intrinsic expressions of *IL-6*, *IL-8* and *XIAP* in both cancer cells. Moreover, the effect of PTX to induce IL-6, IL-8 and XIAP in TLR-4 deficient MDA-MB-231 BCA cells was not statistically significant (Fig. 2a and b).

**Fig. 3:**
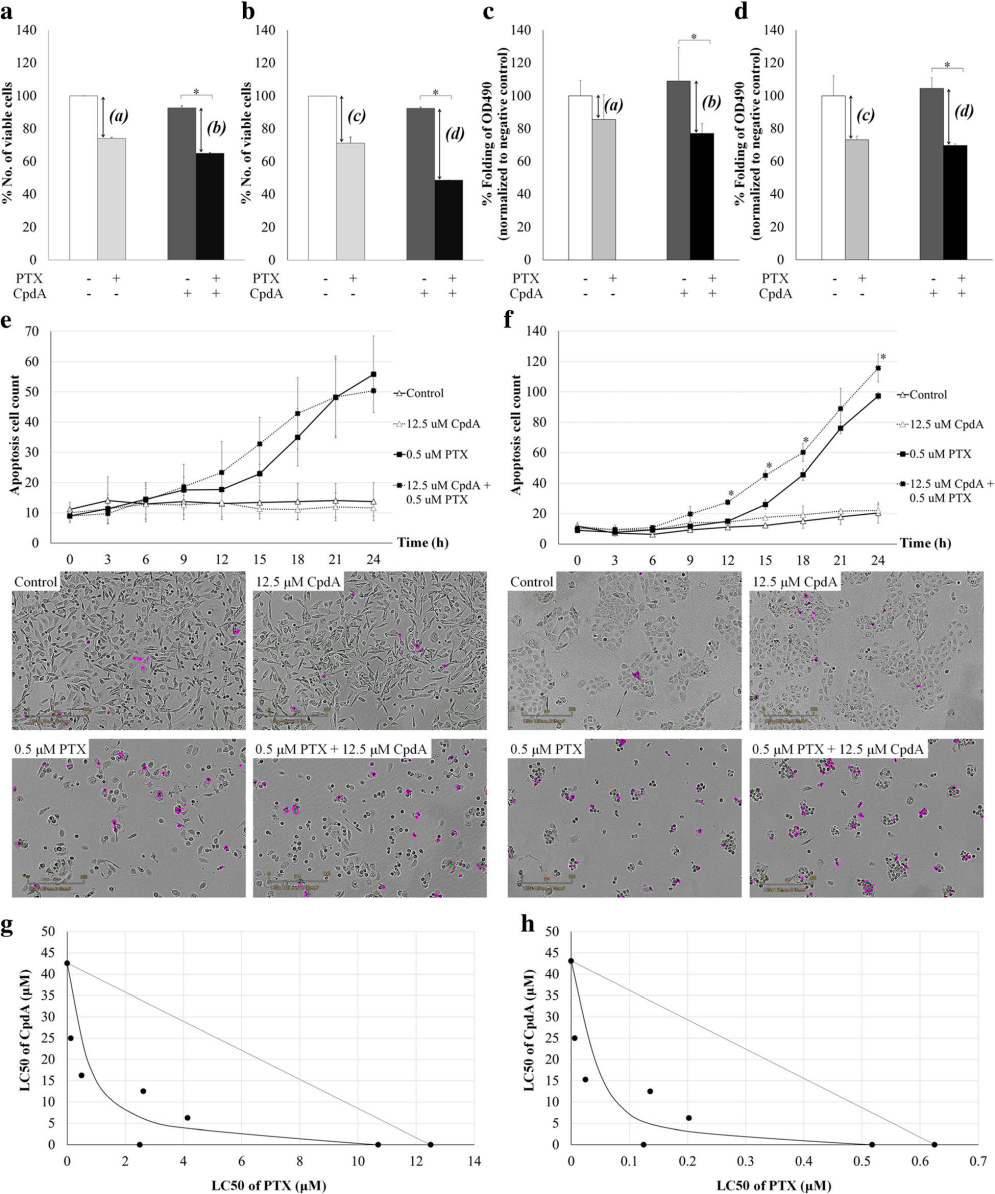
Effect of CpdA on PTX cytotoxicity. The cytotoxicity to PTX in the condition of cells with and without CpdA in (**a** and **c**) MDA-MB-231 and (**b** and **d**) MDA-MB-435. Numbers of viable cells were counted by trypan blue staining in (**a**) and (**b**) and by MTS assay in (**c**) and (**d**). The cell reduction after PTX treatment of CpdA-treated cells was compared to that of parental cells as controls represented as *(a)* and *(b)* for MDA-MB-231; *(c)* and *(d)* for MDA-MB-435 cells. *p-value < 0.05. Graphs represent mean ± SD of apoptotic cell counts identified by caspase-3/7 apoptosis assay at different time points of different treatments for (**e**) MDA-MB-231 and (**f**) MDA-MB-435. The 2–3 independent experiments were performed. *p-value < 0.05 compared to PTX treatment. Microscopic pictures of apoptotic cells are shown in according to the line graphs. Scale bar = 300 μm and original magnification 100X. The isobolograms represent the synergistic effect of CpdA to the cell killing effect of PTX in (**g**) MDA-MB-231 and (**h**) MDA-MB-435

Cell cytotoxicity to PTX with and without CpdA was measured and it was revealed that PTX could cause 26% cell death in MDA-MB-231 BCA [Fig. 3a, *(a)*] and 29% in MDA-MB-435 melanoma cells [Fig. 3b, *(c)*] compared to the untreated condition. In the combined PTX and CpdA treatment, the numbers of viable cells were 30% reduced (*p*-value = 0.026) in MDA-MB-231 [Fig. 3a, *(b)*] and 47% in MDA-MB-435 cells (*p*-value = 0.02) [Fig. 3b, *(d)*] compared to untreated controls. The similar pattern of results were observed when using MTS to measure the viable cells with (Fig. 3c, *p*-value = 0.006 and 3D, *p*-value = 0.018). Moreover, the caspase-3/7 activated cells represented apoptotic cells were increased in both BCA and melanoma cells treated with PTX significantly compared to those in control condition without treatment (Fig. 3e and f). The cancer cells exposed to the combined PTX and CpdA showed significantly increased apoptotic cells compared to PTX-treated condition in MDA-MB-435 (Fig. 3f) and slightly increased in MDA-MB-231 (Fig. 3e). Moreover, the isobologram revealed the increasing effect of CpdA to PTX-induced cell death in the synergistic manner in both MDA-MB-231 and MDA-MB-435 cells (Fig. 3g and h).

**Fig. 4:**
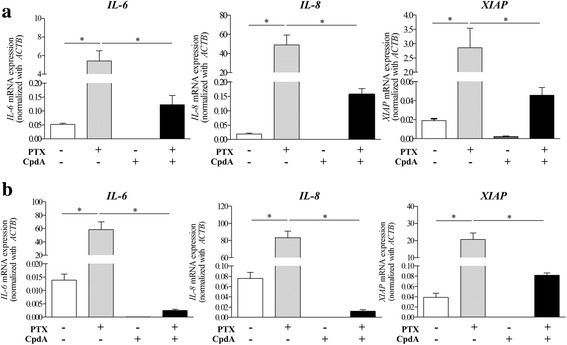
CpdA attenuates IL-6, IL-8 and XIAP expressions in PTX-treated BCA cells. IL-6, IL-8 and XIAP mRNA levels were detected by real time PCT in (**a**) MDA-MB-231 and (**b**) MDA-MB-435. Bars represent mean ± SD of independent duplicate experiments. *p-value < 0.05

**Fig. 5:**
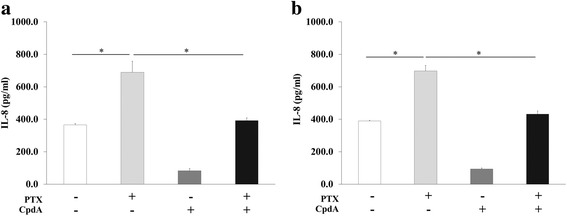
IL-8 level in PTX- and CpdA-treated BCA cells. Protein levels of IL-8 measured by ELISA in (**a**) MDA-MB-231 and (**b**) MDA-MB-435 cells after either 25 nM PTX or 12.5 μM CpdA treatments. Bars represent mean ± SD of independent duplicate experiments. *p-value < 0.05

In addition, CpdA dramatically attenuated TLR4-induced *IL-6*, *IL-8* and *XIAP* expressions in both MDA-MB-231 BCA (Fig. 4A) and MDA-MB-435 melanoma cells (Fig. 4B) compared to cells with only PTX treatment. Using ELISA, IL-8 showed an increasing secreted level compared to PTX untreated controls in both BCA cell lines (Fig. 5a and b). CpdA treatment significantly reduced the secreted IL-8 from both MDA-MB-231 (Fig. 5a) and MDA-MB-435 cells (Fig. 5b).

## Discussion

TLR4 has been previously reported as having high expression in a variety of cancers [18–21] and was found to be involved in tumor proliferation [22], immune escape [18], metastasis [20] and PTX resistance [11–13, 23]. The latter is because PTX has been reported for mimicry to LPS for its binding to TLR4 and to stimulate production of several cytokines [8]. PTX is the microtubule-stabilizing agent used to eradicate tumors by inhibition depolymerization during cell division and induced apoptosis and is typically effective as the second-line drug in BCA patient treatment [24]. In the present study, it was extensively demonstrated that PTX induced pro-inflammatory mediators and tumor survival via TLR4 signaling pathway and CpdA could inhibit this pathway and attenuate the resistance of PTX in BCA and melanoma cells.

In agreement with the report by Wu J. et al. that PTX induced significant augmentation of *TLR4* expression in melanoma cells [5] and TLR4 supported growth and survival of cancer [22, 25], it was found that MDA-MB-231 BCA and MDA-MB-435 melanoma cells treated with PTX had increased expression of *TLR4*, which is similar to the finding in MCF-7 BCA cells in recent publications, that 6-phosphofructo-2-kinase, a critical regulator of glycolysis, modulated *TLR4* expression after PTX exposure [26]. It has been suggested that the PTX-induced TLR4 signaling pathway is through not only NF-κB [11, 13] but also the AP-1 transcription factor could bind to the promoter of *TLR4* gene, leading to TLR4 up-regulation [27]. MDA-MB-231 cells having the highest expression of TLR4 in both mRNA and protein levels compared to MDA-MB-435 and MCF-7 cells, had less cytotoxicity against PTX (higher LC_50_) than MDA-MB-435 cells which was in concordance to TLR4 expression (data not shown). Moreover, blockage of TLR4 and its downstream signaling pathway reversed the effect of TLR4-related PTX resistance. TLR4-deficient cells had more cell death by chemotherapeutic drugs than that of mock cells. MDA-MB-231 with TLR4 depletion had decreased level of pro-survival genes expression and IC_50_ to PTX together with the reduced recurrence rate in vivo [13]. In support, TLR4-transfected cells significantly expressed inflammatory and pro-survival cytokines leading to the resistance to PTX [13]. Taken all together, it suggests that PTX can induce drug resistance in MDA-MB-231 BCA and MDA-MB-435 melanoma cells via a TLR4 dependent pathway.

In the current study, a significant reduction in viable cells of both TLR4-deficient cancer cells and mock cells after PTX treatment was found. Interestingly, the percentage of cell death was higher in TLR4-deficient cancer cells compared to that of mock cells in both MDA-MB-231 and MDA-MB-435 cells. These results suggested that TLR4 reversed PTX-induced cell death leading to the induction of PTX resistance acquired during PTX treatment. It is consistent to the previous studies that stimulation of the TLR4 signaling pathway sustained chemoresistance and cancer progression [11, 13, 18, 23, 28, 29].

Regarding PTX treatment of cancer cells, most inflammatory mediators exhibited altered expressions [9, 11, 13, 30, 31]. PTX could induce NF-κB and cJUN activation to promote IL-6, IL-8, VEGF and MCP-1 productions via a TLR4/MyD88-dependent pathway and resisted drug-induced apoptosis in ovarian cancer [9, 11]. PTX also induced pAKT and XIAP at various time points after treatment. Herein, it was found that PTX-treated MDA-MB-231 BCA and MDA-MB-435 melanoma cells showed upregulation of *IL-6*, *IL-8* and *XIAP* expression compared to untreated cells. In contrast, IL-6, IL-8 and XIAP levels were reduced in TLR4-knockdown cancer cells. It is a similar pattern to the previous report that PTX could increase *IL-6*, *IL-8*, *TNF-α* and *MCP-1* expressions and silencing TLR4 enhanced the attenuation of NF-κB, pAKT, Bcl-2 and Bcl-xL expressions after PTX administration [13]. It is thus proposed that during PTX elimination of cancer cells, it activates inflammatory mediators, in particular IL-6, IL-8 and XIAP from cancer cells, and these cytokines can then aggravate anti-apoptosis via the TLR4 signaling pathway.

The PTX-resistant phenotype in BCA showed the increased multi-drug resistant genes and cytokines including IL-6 and IL-8 compared to PTX-sensitive BCA cells indicating that the PTX resistant phenotype had developed changes in gene expression that might promote cancer progression [32, 33]. In melanoma, the combination of natural anti-tumor agent with PTX resulted in a significant decrease in the production of IL-8 and VEGF, compared with PTX alone [5]. IL-6 has been reported to enhance angiogenesis via the STAT3 pathway [34] to act as a growth factor [35] and to be involved in chemoresistance in cancers [36]. IL-8 is defined as an autocrine growth factor that includes promotion of proliferation, survival, angiogenesis, invasion and metastases in lung cancer [37], cervical cancer [38] and ovarian cancer [39]. In BCA, the IL-8 level significantly increased in patients with more advanced disease correlated to poor survival [40]. In HER2-negative BCA patients, a high level of IL-8 had a significant association with poor recurrence-free survival [41]. This was in concordance to the findings herein that IL-8 was significantly increased in both TLR4-positive and TLR4-deficient BCA cells when exposed to PTX so that IL-8 might be a predictive marker for tumor-promoting aggressiveness in BCA. Moreover, PTX therapy of patients with TLR4-expressing tumors may activate systemic inflammatory circuits that promote metastasis at both local sites and pre-metastatic niches in distal organs. It is recently suggested that blocking TLR4 could significantly improve response to PTX therapy [13] by regulating both local and systemic inflammatory pathways that promote malignant progression, tumor recurrence and the establishment of metastatic lesions, either during chemotherapy or after it is completed [42]. In support to these findings, TLR4-mediated signaling was activated in PTX-resistant melanoma cells and the interfering of this pathway could reverse PTX resistance [5].

XIAP is known as the cellular protein that has evolved to be the most potent inhibitor of the enzymatic activity of mammalian caspases and the promotion of resistance of apoptosis [43]. XIAP has been proposed to inhibit cell death [44] and enhance chemoresistance in cancer [45]. Recently, XIAP has been reported in the PTX-induced TLR4/MyD88-dependent signaling pathway in ovarian cancer [10]. These findings suggested that XIAP was stimulated by PTX via TLR4 signaling pathway. NF-κB is a vital transcription factor that regulates many genes essential for cell growth and differentiation, i.e. cytokines and growth factors. This supports the findings herein that XIAP was up-regulated in PTX-treated BCA and melanoma cells.

**Fig. 6:**
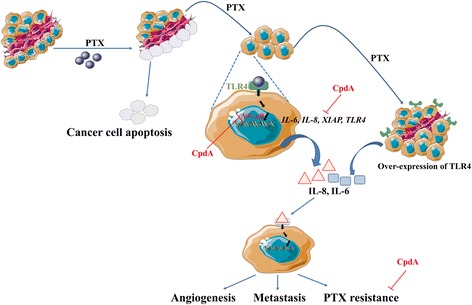
Proposed model of CpdA attenuation of PTX resistance with TLR-4-mediated IL-6, IL-8 and XIAP. TLR4 expressing BCA cells were activated by PTX during PTX administration aiming to kill cancer cells, to release pro-inflammatory cytokines IL-8 and IL-6 into the tumor microenvironment. The mechanism is a TLR-4 and NF-κB dependent pathway. TLR4 is the other target of this activated pathway. The PTX-TLR4 mediated effect can aggravate cancer progression including metastasis, PTX resistance and angiogenesis. This phenomenon can be targeted by a NF-κB specific inhibitor, CpdA, leading to the attenuation of TLR-4-mediated PTX resistance

CpdA was capable of efficiently down-modulating NF-κB and AP-1-driven genes involved in inflammation and cell survival via the glucocorticoid receptor [14, 46, 47]. The current results, under the combined effect of PTX and CpdA, showed that these two drugs had a synergistic effect to support PTX-induced apoptosis in both MDA-MB-231 BCA and MDA-MB-435 melanoma cells. The number of viable cells in the combined PTX and CpdA treatment was lower than that of PTX treatment alone. This is supported by more apoptotic cells significantly observed in MDA-MB-435 than those of PTX treatment. However, this was not significantly in MDA-MB-231 which may be explained by the previous report that CpdA could induce cell to undergo other program cell death such as autophagy [48]. In our study, apoptosis is the majority of cancer cell death induced by PTX and CpdA helps cancer cells to better respond to PTX. It is possible to explain that CpdA treatment provides significantly attenuated inflammatory gene expression induced by PTX including IL-6, IL-8 and XIAP. In good agreement, CpdA efficiently activated trans-repression of NF-κB and AP-1 transcription factors [14, 15, 49] to suppress IL-6 and IL-8 productions [46]. Taken together, it is suggested that TLR4-driven PTX resistance in BCA is via the TLR4/NF-κB signaling pathway through the activation of inflammatory mediators i.e. IL-6, IL-8 and XIAP; the secreted IL-8 is significantly increased after PTX treatment onto BCA cells (Fig. 6). IL-8 (and IL-6) can activate cancer aggressiveness by the induction of cancer metastasis and PTX resistance. The angiogenesis effect of IL-8 may be the other mechanism of cancer progression during PTX treatment. In addition, since the finding that PTX treatment was largely efficacious in inhibiting TLR4-negative tumors, it is not surprised that in PTX treatment, the increased incidence and burden of pulmonary and lymphatic metastasis in TLR4-positive tumors was significant [42]. Therefore, blocking TLR4 signaling at the same time of starting or during PTX treatment by a specific inhibitor such as CpdA may be of benefit to attenuate PTX resistance and/or metastasis in BCA.

Several lines of study to improve the efficacy of PTX and reduce the toxicity of PTX have been performed such as nanoparticle formulation albumin-bound PTX (nab-PTX) in metastatic breast cancer and melanoma [50, 51]. The results of Phase II/III studies indicate that nab-paclitaxel may be effective as neoadjuvant treatment of triple negative BCA [50]. For the treatment of advanced or metastatic melanoma, based on positive Phase II trial data, nab*-*PTX led to response rates of 22% to 26% in chemotherapy-naïve patients with metastatic melanoma [51]. These evidences highlight the importance of PTX in the treatment of advanced BCA and melanoma patents. Knowing the mechanism of how cancer cells resist to PTX and how CpdA can increase the sensitivity of cancer cells would suggest the clinical usage of CpdA as a synergistic drug to sensitize cancer cells to PTX. Moreover, the inhibitors of PTX-TLR4 signaling pathway or the inhibitors of IL-8 can be considered as the alternative treatment to inhibit PTX resistance.

## Conclusion

The resistance to PTX occurs during advanced BCA and melanoma patient treatment. TLR-4/NF-κB dependent pathway can be activated by PTX to promote the survived cancer cells to produce pro-inflammatory cytokines, some of which can stimulate cancer aggressiveness. The impact of the TLR4-mediated pathway to control the production of IL-6 and IL-8 has been shown and that after release into the tumor microenvironment, it can have a paracrine effect on either the neighboring cancer cells to survive or resist to PTX. In addition, with this pathway, the anti-apoptotic protein XIAP is induced in cancer cells leading to the anti-apoptotic response to PTX. CpdA can target this pathway and attenuate TLR4-mediated PTX resistance in BCA and melanoma; hence, combination treatment with PTX and CpdA may be considered not only to sensitize cancer cells to PTX, but also to prevent PTX resistance.

## Additional file

Additional file 1: **Figure S1.** TLR4 and MyD88 expressions of BCA cell lines. (A) Western blot analysis revealed TLR4 in all cells. Equal total protein loading was confirmed by β-ACTIN internal control. Bar graphs represent intensities of TLR4 bands quantified by ImageJ® software and normalized with that of β-ACTIN. (B) Immunocytochemistry for TLR4 in BCA cell lines. TLR4 was labeled with goat anti-human TLR4 and donkey anti-goat IgG-Alexa Fluor® 488 (green). Hoechst® 33,258 (blue) was used for nuclei staining (Scale bar = 20 μm and original magnification 400X). (C) MyD88 was detected by real-time PCR. *MyD88* mRNA expression level was normalized by *ACTB* as an internal control. Bars represent mean ± SD of duplicate PCR reactions. (D and E) LC_50_ of MDA-MB231 and MDA-MB435 against PTX after 24 h incubation is shown. (TIFF 8856 kb)

## Abbreviations

AP-1: activating protein-1
BCA: breast cancer
BCL2L11 or Bim: Bcl-2-like protein 11
Bik: Bcl-2 –interacting killer
cDNA: complementary DNA
CpdA: compound A
DAMP: damage-associated molecular patter
GR: glucocorticoid receptor
IL-6: interleukin 6
IL-8: interleukin 8
LC_50_: 50% lethal concentration
LPS: lipopolysaccharide
MAPK: mitogen-activated protein kinases
MCP-1: monocyte chemoattractant protein-1
mRNA: messenger RNA
MyD88: myeloid differentiation factor 88
NF-κB: nuclear factor kappa-light chain-enhancer of activated B cells
p53: tumor protein 53
PTX: paclitaxel
SD: standard deviation
siRNA: short interfering RNA
TLR4: toll-like receptor 4
VEGF: vascular endothelial growth factor
XIAP: X-linked inhibitor of apoptosis
