# Supplementary figures and images for: Compound A attenuates toll-like receptor 4-mediated paclitaxel resistance in breast cancer and melanoma through suppression of IL-8
Source: BMC Cancer. 2018 Feb 27;18:231. doi: 10.1186/s12885-018-4155-6 (PMC5830047; doi:10.1186/s12885-018-4155-6)

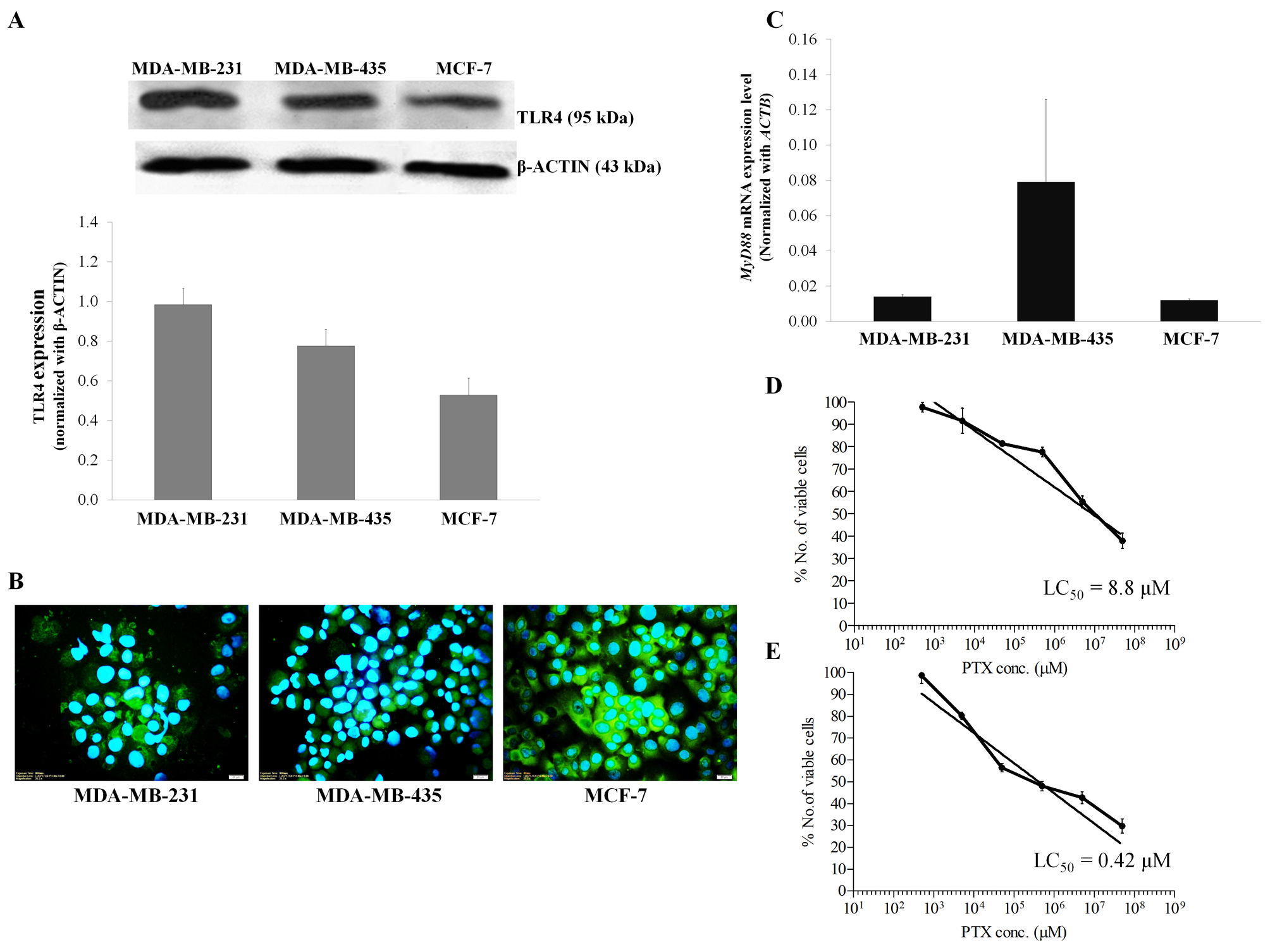

Supplement: Supplementary file 1 — Figure S1. TLR4 and MyD88 expressions of BCA cell lines. (A) Western blot analysis revealed TLR4 in all cells. Equal total protein loading was confirmed by β-ACTIN internal control. Bar graphs represent intensities of TLR4 bands quantified by ImageJ® software and normalized with that of β-ACTIN. (B) Immunocytochemistry for TLR4 in BCA cell lines. TLR4 was labeled with goat anti-human TLR4 and donkey anti-goat IgG-Alexa Fluor® 488 (green). Hoechst® 33,258 (blue) was used for nuclei staining (Scale bar = 20 μm and original magnification 400X). (C) MyD88 was detected by real-time PCR. MyD88 mRNA expression level was normalized by ACTB as an internal control. Bars represent mean ± SD of duplicate PCR reactions. (D and E) LC50 of MDA-MB231 and MDA-MB435 against PTX after 24 h incubation is shown. (TIFF 8856 kb) [file 12885_2018_4155_MOESM1_ESM.tif]
